# Effects of Indigo Carmine on Growth, Cell Division, and Morphology of *Allium cepa* L. Root Tip

**DOI:** 10.3390/toxics12030194

**Published:** 2024-03-01

**Authors:** Madalina-Elena Ristea, Otilia Zarnescu

**Affiliations:** Faculty of Biology, University of Bucharest, Splaiul Independentei 91-95, R-050095 Bucharest, Romania; ristea.madalina-elena@s.bio.unibuc.ro

**Keywords:** food dye, textile dye, E132, cytogenotoxicity, chromosomal anomalies, mitotic index

## Abstract

Indigo carmine has a variety of uses in foods, textiles, medicine, pharmaceuticals, and cosmetics. There are studies reporting the toxic potential of indigo carmine on human health and the environment. In this study, we investigated the cytogenotoxic effects of indigo carmine using apical root cells of *Allium cepa*. *Allium cepa* bulbs were subjected to four treatments with indigo carmine (0.0032, 0.0064, 0.0125, and 0.2 mg/mL) and to ultrapure water as a control. After 5 days, root growth, root length, mitotic index, mitotic inhibition, chromosomal anomalies, and cell morphology were analyzed. According to our results, a decrease in root length and mitotic index was observed at all concentrations of indigo carmine. Additionally, several types of chromosomal abnormalities were observed, such as disturbed metaphase, sticky chain metaphase, anaphase bridge, and laggard chromosomes. Moreover, histological observation indicated that indigo carmine induces alterations in various components of root tip tissue, such as deformation and alteration of the cell wall, progressive condensation of chromatin, shrinkage of the nuclei, and an increase in the number of irregularly shaped nuclei and nuclear fragments. Our results indicate that the tested concentrations of indigo carmine may have toxic effects and raise concerns about its intensive use in many fields.

## 1. Introduction

Indigo carmine is an intensively used dye, but at the same time can be a threat to the environment and human health [1]. Indigo carmine is a dark blue and water-soluble powder known as indigotine, E132, 5,5′-indigodisulfonic acid sodium salt, etc. The chemical formula for indigo carmine is C_16_H_8_N_2_Na_2_O_5_S_2_ [2,3,4,5,6,7,8]. Despite its toxic potential reported in the literature, indigo carmine has many uses in foods, medicine, textiles, pharmaceuticals, and cosmetics [6,9,10,11,12,13,14].

Although adverse effects such as nausea, vomiting, diarrhea, and skin irritation have been reported following ingestion, the dye is found in many foods, such as bubble gum, jellies, desserts, beverages, etc. [7,15]. The European Food Safety Authority allows the use of indigo carmine in food and beverages with maximum permitted levels between 50 and 500 mg/kg [1,16], while the Codex Alimentarius Commission has approved maximum levels ranging from 50 to 450 mg/kg [1,17]. The U.S. Food and Drug Administration (USFDA) has not established maximum levels for this food additive but allows its use under good manufacturing practices in a number of foods as listed in the Code of Federal Regulations [1,18].

Indigo carmine has many uses in medicine: in orthopedics and trauma surgery for staining cystic lesions or herniated disc surgery [19,20], in microsurgery, vasography, vasovasostomy, vasoepididymostomy, gynecological laparoscopy [21,22,23], in angiography [24], in colonoscopy [25,26], etc. Although indigo carmine is reported to be a biologically inactive dye, there are many adverse effects associated with its intravenous administration, such as hypertension [27,28,29,30,31], bradycardia, and arrhythmias [21,30]. Severe irritation, edema, or discoloration may also occur following skin exposure [7,30,32,33].

Because indigo carmine is heavily used in the textile industry for dyeing denim and polyester fibers, it is often discharged into natural water sources [6]. Therefore, dyes such as indigo carmine cause environmental pollution and are a threat to aquatic organisms and human health [10,34,35]. It can increase water turbidity, affect plant photosynthesis, and accumulate in aquatic organisms [36]. In addition, water contaminated with indigo carmine changes color and odor and becomes non-potable even at very low concentrations [1].

The effects of indigo carmine have been tested in vivo on several species, such as *Mus musculus* [37,38], *Sus domesticus* [39], and *Eisenia andrei* [7], and in vitro on cell cultures such as chondrocytes [20] and fibroblasts [13]. Studies on the effects of indigo carmine on plants have been scarce and have been conducted so far only on two species, the biennial herbaceous monocotyledon plant *Allium cepa* [40] and the green alga *Chara vulgaris* [41].

The Allium test is used to evaluate the genotoxic potential of various types of chemicals and biological agents on numerous plant species [42,43]. Generally, plants play an essential role as bioindicators because they are sensitive to changes in the environment and react to numerous stimuli by changing their morphology. Also, the genetic structure of plants is useful for testing the toxic effects of chemical substances and contaminants. The International Program on Plant Bioassay (IPPB) of the United Nations Environmental Program (UNEP) and Gen-Tox of the U.S. Environmental Protection Agency (USEPA) validated the use of the Allium test as a bioindicator. Also, USEPA and the World Health Organization (WHO) considered that the results obtained from the Allium test are reliable and effective in detecting environmental genotoxicity in water, air, and soil [43,44].

The most frequently used plants for toxicity testing are *Allium cepa* L., *Lens culinaris* Med [42,45,46], *Pisum sativum* L [47], *Cladonia verticillaris* Raddi, *Lactuca sativa* L. [48] and *Vicia faba* L. [49]. In general, plants belonging to the genus Allium, especially *Allium cepa*, are considered the most useful and effective for genotoxicity assessment due to their advantages [42,50]. Among the many advantages are low number of chromosomes (*Allium cepa* 2n = 16), large chromosomes, large genomes (>15,000 Mbp) [51], roots that can grow in direct contact with any tested substance, easy identification *of* mitotic phases and chromosomal aberrations [52], rare occurrence of spontaneous chromosomal lesions, fast response to genotoxic compounds, diversity in chromosome morphology, low cost, and easy to handle [50,53,54].

The Allium test can determine several cytogenic and genotoxic parameters, such as mitotic index, phase indices, chromosomal aberrations, mitotic abnormalities, and micronuclei formation [44,50,54,55,56]. Chromosomal aberrations and micronuclei formation in meristematic cells of *Allium cepa* have been used as reliable biological indicators and predictive markers for xenobiotic-induced DNA damage [45,46,54,56,57]. The Allium test has been reported to be sensitive and can detect the genotoxic and mutagenic potential of any natural, chemical, or synthetic compound. Allium test results can be correlated with other test systems such as algae, fish, bacteria, animal, and even human cell culture testing [44,50,54,55,56]. Also, the *Allium cepa* chromosomes share morphological similarities with those of mammals [57]. In addition to microscopic observations of cytogenetic effects, xenobiotics toxicity can be assessed macroscopically through the evaluation of root growth inhibition [56]. The Allium test has been used to detect the effects of many chemicals and environmental pollutants, and the results obtained were satisfactory to the majority of the reported studies. Among the substances tested on *Allium cepa* are dyes, metals, pesticides, and aromatic hydrocarbons [54]. Also, the Allium test has been used to identify the genotoxicity of some food dyes: metanil yellow, carmoisine [58], allura red (E129), sunset yellow (E110), fast green (E143), and tartrazine (E102) [59]. Therefore, the Allium test is a valuable in vivo model to study the cytotoxic and genotoxic effects of pollutants in water, soil, and air, as well as other chemical substances [51,53,60].

In this study, we evaluated the cytotoxic and genotoxic effects of indigo carmine on *Allium cepa* by measuring root size, calculating the mitotic index and mitotic inhibition, and determining the percentage of abnormal mitoses. The effects of this dye on the root tip histology were also investigated. To the best of our knowledge, no similar studies have been reported.

## 2. Materials and Methods

### 2.1. Chemicals

Indigo carmine (E132) was purchased from Sigma-Aldrich (St. Loius, MO, USA) and is certified by the BSC (Biological Stain Commission). Four different dye concentrations (0.0032 mg/mL, 0.0064 mg/mL, 0.0125 mg/mL, and 0.2 mg/mL) prepared in Milli-Q ultrapure water (Merck, Milli-Q Direct 8 Water Purification System, Molsheim, France) were used in this study.

### 2.2. Plant Material

The onion bulbs (*Allium cepa* L.: 2n = 16) used in our experiment were of the Stuttgarter variety. They were procured from an agricultural farm that does not use chemical herbicides, fungicides, or fertilizers. Healthy onion bulbs of similar size, with an average weight and diameter of 14.21 ± 1.7 g and 3.77 ± 0.2 cm, respectively, were chosen for the experiment.

### 2.3. Experimental Design

Before starting the experiment, brown leaves were removed from the surface of *Allium cepa* bulbs so that the root primordia was not affected. Subsequently, *Allium cepa* bulbs were placed in 50 mL Falcon tubes so that only the base of the bulb was immersed in the liquid. For each concentration of indigo carmine and the control group, three bulbs of *Allium cepa* were used. For the control group, *Allium cepa* bulbs were immersed in ultrapure water. The experiment was performed under controlled environmental conditions and lasted 5 days. An average temperature of 25.3 ± 0.4 °C was maintained during the experiment and the bulbs were protected from direct sunlight. To avoid liquid evaporation, the solutions were changed every 24 hours. Subsequently, after 5 days, the grown roots were sectioned, measured with a graduated ruler, and counted.

The root growth of *Allium cepa* in control and after exposure to indigo carmine was calculated as follows:

$$Root\;growth\;(\%)=\frac{Mean\;root\;length\;of\;treatments}{Mean\;root\;length\;of\;control}\times 100$$

The Feulgen-squash method was used to prepare slides for analyzing root meristematic cells [43,55,57,61,62]. Apical meristems were washed in distilled water and then fixed in Carnoy [glacial acetic acid:ethanol (1:3)]. To observe mitotic stages and chromosomal anomalies, apical meristems were hydrolyzed in 1N HCl at 60 °C for 5 min and stained with 1% aceto-orcein for 15 min. Afterward, the tip of the meristem was placed on a glass slide, covered with a coverslip, and crushed. The slides obtained were analyzed under a microscope to calculate the mitotic index, mitosis inhibition, and chromosomal aberrations. The mitotic activity was analyzed by looking at the morphological changes in the nuclei during cell division and structural chromosomal aberration.

The mitotic index was calculated according to the formula [63]:

$$Mitotic\;index\;(MI)=\frac{dividing\;cells\;in\;all\;phases}{total\;number\;of\;cells}\times 100$$

The mitotic inhibition can be calculated as [50]:

$$Mitotic\;inhibition=\frac{MI\;in\;the\;control\;group-MI\;in\;the\;test\;group}{MI\;in\;the\;control}\times 100$$

### 2.4. Analysis of Cell Morphology Using Fluorescent Microscopy

To analyze cell morphology, *Allium cepa* root tips were processed for embedding in paraffin. The root tips were fixed in 4% formalin in PBS, dehydrated by immersing in a series of ethanol solutions of increasing concentrations (70–100%), cleared in toluene, and then embedded in paraffin. The 6-μm thick sections were stained with 0.05% Acridine orange in PBS and 1% Fast green FCF in 3% acetic acid. The sections were examined with a Zeiss Axiostar Plus microscope (Zeiss, Oberkochen, Germany). The photomicrographs were taken using a digital camera (AxioCam MRc 5; Zeiss) driven by the AxioVision 4.6 software (Zeiss).

### 2.5. Data and Statistical Analysis

Results were expressed as mean values ± standard deviation. GraphPad Prism 5 Software (GraphPad Software Inc, San Diego, CA, USA) was used for data interpretation. Comparisons of quantitative variables were performed using a one-way analysis of variance followed by the Bonferroni multiple comparison post hoc test. Significant statistical differences were considered at *p* < 0.05. Levels of significance were indicated as * *p* < 0.05 and ** *p* < 0.001.

## 3. Results

### 3.1. Root Growth

According to the results presented in Table 1, the root length decreased with increasing concentration of indigo carmine. Root measurement indicated statistically significant differences (*p* < 0.05; *p* < 0.001) between the root length of onion bulbs from the control group and those exposed to different concentrations of indigo carmine (Table 1). Therefore, the highest average root length was observed in the control group (1.60 cm), followed by the indigo carmine concentrations of 0.0032 mg/mL (1.42 cm), 0.0064 mg/mL (1.31 cm), 0.0125 mg/mL (1.19 cm), and 0.2 mg/mL (1.04 cm).

Moreover, the percentage of root growth decreases with increasing dye concentration. The percentage of root growth was 100% in the control group, followed by a progressive decrease in the onion bulbs exposed to different concentrations of indigo carmine. A root growth percentage of 88.75% was observed at a concentration of 0.0032 mg/mL, followed by growth percentages of 81.84% at 0.0064 mg/mL, 74.37% at 0.0125 mg/mL, and 65% at 0.2 mg/mL (Table 1).

### 3.2. Mitotic Index and Mitotic Inhibition

The concentrations of indigo carmine used in this experiment had an effect on mitosis as a significant decrease in the mitotic index as well and a significant percentage of mitosis inhibition was observed in the treated groups compared to the control group. The highest value of the mitotic index was observed in the control group (11.45 ± 6.18), while the lowest values were observed at concentrations of 0.0064 mg/mL (5.28 ± 2.95) and 0.2 mg/mL (5.3 ± 2.22) indigo carmine (Table 2). Significant differences were observed between the control and the test groups, but the decrease in mitotic index was not dependent on the increase in dye concentration. Although the mitotic index does not decrease in a dye concentration-dependent manner, no significant differences were observed between the tested groups.

Also, mitosis inhibition was between 53.88% at a concentration of 0.0064 mg/mL and 47.16% at a concentration of 0.0125 mg/mL. Mitotic inhibition of 53.71% was observed at an indigo carmine concentration of 0.2 mg/mL. Similar to the mitotic index, no correlation was observed between mitosis inhibition and increased dye concentration, and no significant differences were observed between the tested groups.

### 3.3. Chromosomal Anomalies

**Figure 1:**
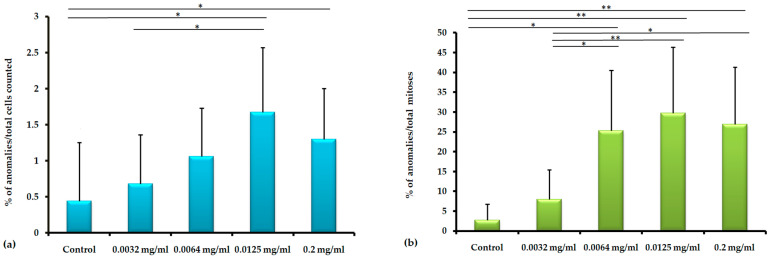
Chromosomal anomalies: abnormal metaphases + abnormal ana/telophases in *Allium cepa* after 5 days of exposure to water (control) and in the presence of 0.0032, 0.0064, 0.0125, and 0.2 mg/mL of indigo carmine. (**a**) Percentage of anomalies reported versus the total cells counted; (**b**) Percentage of anomalies reported versus the total number of mitoses. Bars represent standard deviation. Levels of significance were indicated: * significant (*p* < 0.05); ** highly significant (*p* < 0.001).

In our study, a higher percentage of chromosomal anomalies were observed in roots exposed to indigo carmine concentrations in contrast to the control group. The total percentage of chromosomal anomalies is shown in Figure 1. Figure 1a shows the percentage of chromosomal anomalies reported versus the total number of cells, and Figure 1b shows the percentage of chromosomal anomalies reported versus the total number of mitoses. No differences were observed between the results obtained after reporting on the total number of cells and the total number of mitoses. For instance, chromosomal anomalies reported versus the total number of cells have a percentage of 1.6% at the 0.0125 mg/mL concentration and 1.3% at the 0.2 mg/mL concentration, while chromosomal anomalies reported versus the total number of mitoses have a percentage of 29.7% at the 0.0125 mg/mL concentration and 26.96 mg/mL at the 0.2 mg/mL concentration. Although the highest percentage of chromosomal anomalies was recorded at the 0.0125 concentration, no statistically significant differences were observed between the 0.0125 and 0.2 concentrations (Figure 1).

The analysis of mitotic phase frequencies is shown in Table 3. The percentage of prophase was not significantly influenced by the concentration of indigo carmine. For instance, the highest percentage of prophase was observed at a concentration of 0.0032 mg/mL, followed by the control group. Indigo carmine induced a decrease in the percentage of normal metaphases, but no correlation was observed between the dye concentration and the percentage of normal metaphases. The percentage of abnormal metaphases increased in treatment groups, reaching the highest value at 0.0064 mg/mL (25.35 ± 15.19). Percentages of normal ana/telophases were not influenced by the indigo carmine concentration, except at a concentration of 0.0032 mg/mL (7.53 ± 9.29). Similar to abnormal metaphases, it was observed that abnormal ana/telophases increased with increasing concentrations of indigo carmine with the exception of 0.0064 mg/mL, where no abnormal ana/telophase was observed.

**Figure 2:**
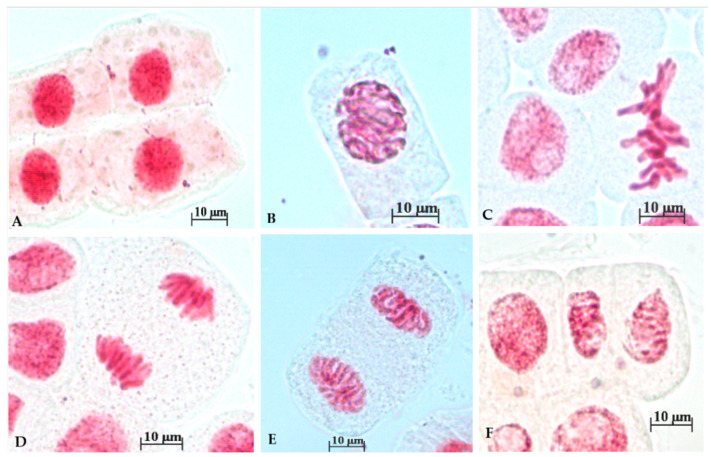
Representative images of individual phases of mitosis in *Allium cepa* root tip cells observed in the control group: (**A**) Interphase; (**B**) Prophase; (**C**) Metaphase; (**D**) Anaphase; (**E**) Late Anaphase; (**F**) Telophase.

Figure 2 shows the expected normal phases of cell division in the control group, such as interphase (A) prophase (B), metaphase (C), anaphase (D, E), and telophase (F).

**Figure 3:**
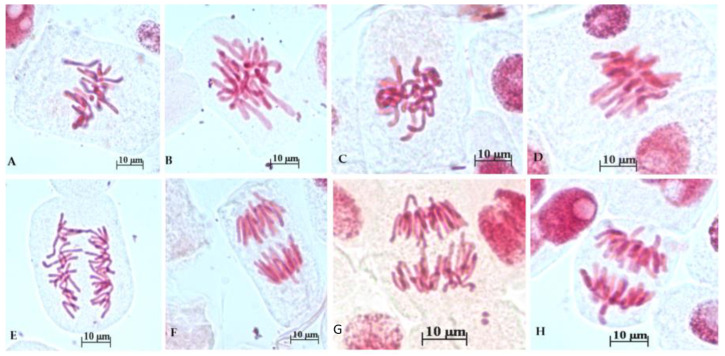
Representative images of chromosomal anomalies observed in *Allium cepa* treated with different concentrations of indigo carmine (IC): Disturbed and sticky chain metaphase: (**A**) 0.0032 mg/mL IC); (**B**) 0.0064 mg/mL IC; (**C**) 0.0125 mg/mL IC; (**D**) 0.2 mg/mL; Anaphase bridge and laggard chromosomes: (**E**) 0.0032 mg/mL IC); (**F**) 0.0064 mg/mL IC; (**G**) 0.0125 mg/mL IC; (**H**) 0.2 mg/mL.

Figure 3 shows representative cytological anomalies. Chromosomal anomalies observed in treatment groups included disturbed and sticky chain metaphase (A–D) and anaphase bridge and laggard chromosomes (E–H).

### 3.4. Analysis of Cell Morphology Using Fluorescent Microscopy

**Figure 4:**
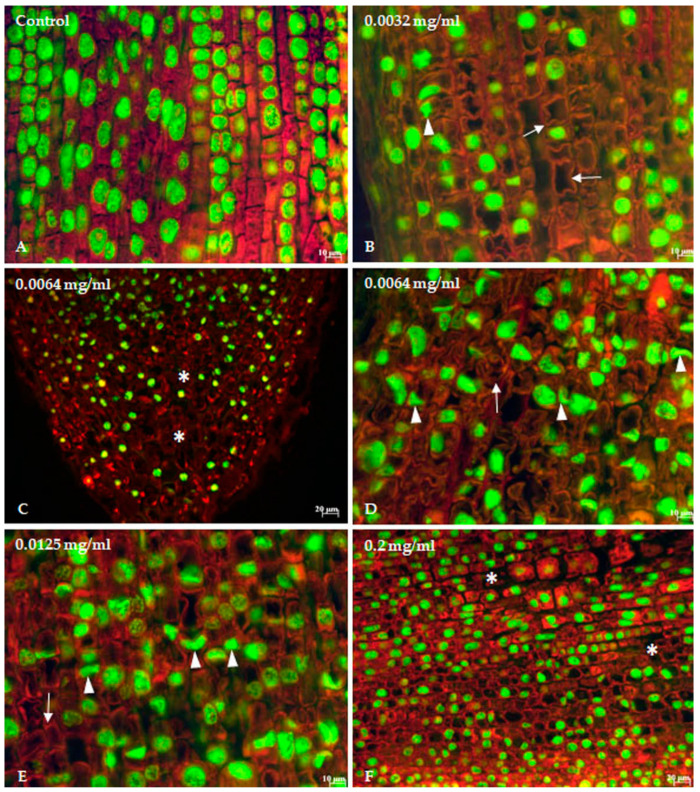
Longitudinal sections of onion root tips observed under a fluorescence microscope demonstrate alterations of cell morphology by indigo carmine. (**A**) Control; (**B**) 0.0032 mg/mL IC); (**C**,**D**) 0.0064 mg/mL IC; (**E**) 0.0125 mg/mL IC; (**F**) 0.2 mg/mL. Morphological alterations include deformed and disrupted cell walls (arrows), irregularly shaped nuclei and nuclear fragments (arrowheads), and chromatin condensation. Abnormal spaces of various sizes were present in root tissue as a result of cell damage (asterisks).

Longitudinal sections of control root tips displayed the organization of the monocotyledon plant and can be divided into four zones: root cap, apical meristem, zone of elongation, and zone of maturation and differentiation. The cells arranged in columns, or files, parallel with the long axis of the root have a homogeneous cytoplasm and a large nucleus with a central location. Cell walls are square or rectangular, and are responsible for the shape of the cells. After staining with Acridine orange and Fast green FCF, cells exhibited reddish, fluorescent cytoplasm and green, fluorescent nuclei (Figure 4A).

Histological observations showed that onion roots exposed to indigo carmine exhibit a progressive disruption of regular cell files and alterations in the cell arrangement become more pronounced as the concentration of indigo carmine increases (Figure 4B–F). As a result of cell damage in the treated roots (Figure 4B–F), abnormal spaces of various sizes were present in root tissue (Figure 4C,F). Moreover, the cytoplasm lost its homogeneous appearance, showed vacuolization, and the cell wall was deformed and disrupted (Figure 4B–F). Treatment with indigo carmine caused progressive condensation of chromatin, shrinkage of the nuclei, and an increase in the number of irregularly shaped nuclei and nuclear fragments (Figure 4B–F).

## 4. Discussion

The aim of our experiment was to determine the cytogenotoxic effects of indigo carmine using meristematic cells of *Allium cepa*. As far as we know, this is the first report describing the effects of indigo carmine exposure on growth, mitotic index, mitotic inhibition, and cell morphology of onion root tips. Roychoudhury and Giri [40] reported the incidence of mitotic aberrations by testing three concentrations of indigo carmine on *Allium cepa* (200, 500, and 1000 ppm), while Bhattacharjee et al. [41] calculated the mitotic index after exposure of the alga *Chara vulgaris* to five concentrations of indigo carmine (100, 250, 500, 750, and 1000 ppm).

The concentrations used in this experiment are within the maximum permitted levels according to the European Food Safety Authority and the Codex Alimentarius Commission. For instance, the concentration of 0.2 mg/mL is equivalent to the concentration of indigo carmine in candied fruit, candies, nougats, fine bakery wares, aromatized alcoholic beverages, etc. [1]. However, in our study, we also considered lower concentrations of indigo carmine because the toxic effect of a substance also depends on the exposure time, and our experiment lasted only 5 days. To the best of our knowledge, the concentration of indigo carmine in wastewater is not exactly established because it depends on many factors, such as the amount of indigo carmine discharged, the effectiveness of the wastewater treatment system, the amount of water, etc.

Root growth occurs as apical meristematic cells pass through interphase and mitosis phases to complete the cell cycle [64]. As a result, the rate of root growth and the mitotic index can be used to measure the toxic impact of a substance on cell proliferation [43,64]. According to recent observations, initially, the toxicity of a compound becomes macroscopically evident as it primarily affects root growth, and the toxic effect is also only observed microscopically [61]. In our study, it was observed that the control group has the highest average root size, and their size decreases with increasing concentration of the tested substance. Similar results were observed after exposure of *Allium cepa* roots to methyl orange [64], titanium dioxide [62], propanil [46], and effluents from a tannery [61].

According to the study conducted by Khanna and Sharma [65], a 45% decrease in root growth percentage indicates genetic alterations in plants due to toxic substances. In our study, this decrease was observed only at a concentration of 0.2 mg/mL. Therefore, inhibition of root growth can be used to demonstrate the cytotoxicity of substances [45,66,67]. Also, growth inhibition can be caused by a reduction in mitotic activity as well as the appearance of several chromosomal aberrations [45,66].

To determine the degree of genetic alterations, it is necessary to take into account the exposure time of the roots to toxic substances [45]. In our study, *Allium cepa* was exposed to the dye for 5 days, similar to the studies by Alaguprathana et al. [64] and Adams and Kerisew [61]; however, the literature contains many studies where onion bulbs are exposed to toxic substances for less time, such as 72 h [46,62], 48 h [43], and even less than 24 h [40,41,68].

Generally, root growth and mitotic index are closely related because both root growth and development depend on the increase in the number of cells, and the mitotic index shows the frequency of dividing cells [67]. The level of cytotoxicity of a substance can be determined based on the increase or decrease in the mitotic index [45,69]. In our study, the mitotic index decreased in the treatment groups compared with control group plants. Similar results were reported by Bhattacharjee et al. [41] after exposure of the green alga *Chara vulgaris* to indigo carmine for 6 h. Also, a decrease in the mitotic index in treatment groups after exposure to different toxics has been reported previously [43,45,54,58,61,64,69,70,71,72,73,74,75]. In our study, no dependence was observed between the concentration of indigo carmine and the effects on the mitotic index or mitotic inhibition. Our results are in agreement with a previous study on the effect of indigo carmine [41].

A significantly lower mitotic index than the control may indicate abnormal changes due to impaired growth and abnormal development of exposed organisms [54]. If the mitotic index decreases by 22% compared to the control group, then the tested substance is considered to have lethal effects, and if the decrease in the mitotic index is 50%, then the substance is considered cytotoxic [69,76]. In our study, the mitotic index decreased by more than 22% compared to the control group at all dye concentrations. Also, a decrease in the mitotic index is caused by the inhibition of DNA synthesis or blocking of the G2 phase of the cell cycle, thus cells cannot start mitosis [64,70].

The study of chromosomal anomalies is another parameter used to determine the cytogenotoxic potential of the tested substances [77]. In our study, chromosomal anomalies were observed during metaphase, anaphase, and telophase. Chromosomal abnormalities are considered a consequence of unrepaired single-strand or double-strand DNA breaks [62]. Chemicals can produce genotoxic effects directly or indirectly by inhibiting the DNA repair system. Abnormalities induced by the direct genotoxic effect can be repaired by DNA repair mechanisms to maintain genome integrity, while indirect genotoxic effects usually prove to be exponentially toxic [43]. In our study, it was observed that indigo carmine can cause an increase in chromosomal aberrations, especially concentrations higher than 0.0125 mg/mL and 0.2 mg/mL. Roychoudhury and Giri [40] reported micronuclei and chromosome breaks after exposure of *Allium cepa* to 1000 ppm indigo carmine for 24 h. In our study, the meristematic root cells of Allium cepa did not exhibit micronuclei after exposure to indigo carmine. Also, in our study, the highest percentage of chromosomal anomalies was recorded at a lower concentration of indigo carmine (0.0125), in contrast to the study by Roychoudhury and Giri [40], where they observed abnormalities only at the highest tested concentration (1000 ppm). The concentration tested by them is higher than those tested in our study. Moreover, in our study, no statistically significant differences in the percentage of anomalies were observed between the 0.0125 and 0.2 concentrations.

Additionally, our results are in agreement with previous reports which showed the toxic effects of other food dyes. A significant increase in chromosomal aberrations was observed following the testing of sunset yellow dye [58,78] and carmoisine [58]. In our study, we observed disturbed and sticky chain metaphase, anaphase bridge, and laggard chromosomes. Sticky chromosomes are considered clastogenic aberrations associated with cell death [54,67] and they are associated with chromosomal bridges, which allow the separation and free movement of chromosomes, thus resulting in connected bridges, breakage, and fusion of chromosomes and chromatids [76]. The appearance of chromosomal bridges highlights the effects of toxic agents on DNA and they can be considered clastogenic alterations [54,67].

Laggard chromosomes are the result of the disruption of the spindle formation process under the action of some toxic agents [79]. Laggard chromosomes move to either side of two poles without attachment of spindle fibers. A cell is considered abnormal even if only one chromosome is damaged or affected [76].

Other observations in our study indicate that indigo carmine induces the alteration in various components of root tip tissue, such as deformation and alteration of the cell wall, progressive condensation of chromatin, shrinkage of the nuclei, and an increase in the number of irregularly shaped nuclei and nuclear fragments. To the best of our knowledge, no data has been reported on indigo carmine effects on plant root tip histology. However, a number of studies have shown that the plant cell wall undergoes a series of changes in response to internal and external factors, including xenobiotics such as metals and metalloids [80,81,82,83], phenoxyethanol [84], manganese [85], and tungsten [86]. Similar ultrastructural changes in the shape of cell nuclei and condensation of chromatin have been reported for onion roots exposed to extracts of *Taxus baccata* shoots, extracts of *Rhodiola rosea* rhizomes [87], and sodium selenate [88]. Nuclear deformation could be associated with the pressure of vesicles or vacuoles that appear as a result of interactions with the tested substances. These changes could also affect the function of the cytoskeleton that maintains the normal shape of cells. Also, nuclear deformation is accompanied by increased chromatin condensation. The larger number of vacuoles in the cytoplasm is the response of the cells when a substance infiltrates the cells due to the detoxification mechanism of plants that produces additional apoplast spaces for storage of non-essential substances or even toxic substances [88,89].

## 5. Conclusions

The Allium test is a useful indicator of cytotoxicity and genotoxicity of various types of chemicals and biological agents, and the results can be correlated with other test systems. In our study, after indigo carmine exposure, *Allium cepa* roots showed a decrease in length, as well as a decrease in the mitotic index. It was also observed that higher dye concentrations produce a higher percentage of chromosomal anomalies, such as disturbed metaphase, sticky chain metaphase, anaphase bridge, and laggard chromosomes. Histological observations indicate that roots exposed to indigo carmine exhibit alterations in cell arrangement and the cytoplasm has a less homogeneous appearance, with vacuoles and cell walls being deformed and disrupted. Moreover, exposure to indigo carmine caused shrinkage of the nuclei and an increased number of irregularly shaped nuclei. Therefore, these results indicated that indigo carmine exerts a cytotoxic and genotoxic impact on meristematic cells in *Allium cepa.* Since indigo carmine is extensively used in many fields, further analysis needs to be conducted to evaluate its toxic potential.

## Figures and Tables

**Table 1:** Root growth and root length of *Allium cepa* roots were determined after 5 days of exposure to water (control) and the presence of 0.0032, 0.0064, 0.0125, and 0.2 mg/mL of indigo carmine.

Concentration	Root Growth (%)	Root Length (cm)
Control	100%	1.60 ± 0.51
0.0032 mg/mL	88.75%	1.42 ± 0.46 **
0.0064 mg/mL	81.87%	1.31 ± 0.31 **
0.0125 mg/mL	74.37%	1.19 ± 0.38 **
0.2 mg/mL	65%	1.04 ± 0.48 **

Values are expressed as mean ± standard deviation. Level of significant differences compared with the control group: ** highly significant, *p* < 0.001.

**Table 2:** Mitotic index and mitotic inhibition of *Allium cepa* were determined after 5 days of exposure to water (control) and the presence of 0.0032, 0.0064, 0.0125, and 0.2 mg/mL of indigo carmine.

Concentration	Mitotic Index (Mean ± Standard Deviation)	Mitotic Inhibition (%)
Control	11.45 ± 6.18	-
0.0032 mg/mL	5.5 ± 3.8 **	51.96%
0.0064 mg/mL	5.28 ± 2.95 **	53.88%
0.0125 mg/mL	6.05 ± 2.4 **	47.16%
0.2 mg/mL	5.30 ± 2.22 **	53.71%

Values are expressed as mean ± standard deviation. Level of significant differences compared with the control group: ** highly significant, *p* < 0.001.

**Table 3:** Cytological analysis of *Allium cepa* in the control and after 5 days of exposure to 0.0032, 0.0064, 0.0125, and 0.2 mg/mL of indigo carmine. Normal and abnormal mitotic phases (prophases, metaphases, and ana/telophases) were expressed as mean values ± standard deviation.

Concentration	% Prophases	% Normal Metaphases	% Abnormal Metaphases	% Normal Ana/Telophases	% Abnormal Ana/Telophases
Control	60.94 ± 14.84	15.15 ± 8.66	0.90 ± 1.85	21.85 ± 9.56	1.12 ± 2.3
0.0032 mg/mL	79.70 ± 11.93	3.88 ± 7.81	5.59 ± 7.08	7.53 ± 9.29	2.16 ± 4.34
0.0064 mg/mL	51.53 ± 24.58	3.35 ± 7.7	25.35 ± 15.19	19.74 ± 25.24	0
0.0125 mg/mL	42.96 ± 14.26	7.96 ± 10.40	20.78. ± 19.45	19.35 ± 13.64	9.02 ± 4.25
0.2 mg/mL	47.77 ± 11.31	7.45 ± 9.72	8.24 ± 8.99	17.31 ± 12.03	19.25 ± 11.67

## Data Availability

Data are contained within the article.
